# Quality of Life and Functional Outcomes Among Post‐Stroke Patients in Al‐Baha, Saudi Arabia: Associations With Demographic, Clinical, and Rehabilitation Factors: A Cross‐Sectional Study

**DOI:** 10.1002/hsr2.71616

**Published:** 2025-12-02

**Authors:** Yasser M. Kofiah, Turki Alkully, Batol M. Albanghali, Haya A. Alzahrani, Shahad M. Alghamdi, Atheer N. Alzahrani, Rana A. Algahamdi, Abdulrahman M. Alzahrani, Uthman Albakri, Basim A. Othman, Mohammad A. Albanghali

**Affiliations:** ^1^ Department of Surgery, Faculty of Medicine Al‐Baha University Al Baha Saudi Arabia; ^2^ Department of Internal Medicine, Faculty of Medicine Al‐Baha University Al Baha Saudi Arabia; ^3^ Faculty of Medicine Al‐Baha University Al Baha Saudi Arabia; ^4^ Department of Internal Medicine King Fahad Hospital Al Baha Saudi Arabia; ^5^ Department of Public Health Faculty of Applied Medical Sciences Al‐Baha University Al Baha Saudi Arabia

**Keywords:** EQ‐5D‐3L, post‐stroke, quality of life, Saudi Arabia, Stroke

## Abstract

**Background and Aim:**

Stroke remains one of the leading causes of long‐term disability worldwide, profoundly impacting patients' quality of life (QoL) and functional independence. This study aimed to assess quality of life (QoL) and functional outcomes among post‐stroke patients in Al‐Baha, Saudi Arabia, and to examine their associations with demographic, clinical, and rehabilitation factors.

**Methods:**

This study assessed QoL and functional outcomes among 125 post‐stroke patients in Al‐Baha, Saudi Arabia, from April 2023 to May 2024 at King Fahad Hospital. Quality of life (QoL) was evaluated using the EuroQol 5‐Dimension 3‐Level (EQ‐5D‐3L), while functional outcomes were measured with the Modified Rankin Scale (mRS). Associations of sociodemographic and clinical variables with QoL and functional outcomes were analyzed using chi‐square tests.

**Results:**

The mean QoL score was 14 ± 4.57, indicating moderate impairment. Mobility and pain/discomfort were the most impacted dimensions, with 12% and 37% of participants reporting extreme issues, respectively. Self‐rated health revealed that 45% of participants considered themselves ‘excellent’ health, while 19% rated their health as ‘poor’ (*p* < 0.001). Younger participants (18–30 years) reported better QoL (*p* = 0.025). Functional outcomes revealed that 59% had favorable mRS scores (0–2), while 41% had poor outcomes (3–6). Physiotherapy utilization was associated with functional outcomes, but poorer outcomes were observed among recipients likely due to selection bias. (*p* = 0.047) but not with QoL scores.

**Conclusions:**

Findings highlight mobility limitations and pain/discomfort as the most affected dimensions of quality of life. Younger patients demonstrated better outcomes, while physiotherapy was associated with functional status, likely reflecting baseline severity rather than treatment effect. These results emphasize the importance of targeted, individualized rehabilitation strategies for post‐stroke populations.

## 1 Introduction

Stroke is one of the leading causes of disability and death globally, with an estimated 12.2 million new cases and 6.5 million related deaths annually [1]. It significantly affects survivors’ quality of life (QoL) and functional independence, leading to substantial physical, psychological, and social burdens. While the global prevalence of stroke is well‐documented, regional variations in risk factors, management, and rehabilitation outcomes have been widely reported, underscoring the need for localized research to design effective interventions [2, 3, 4]. In Saudi Arabia, lifestyle changes—particularly increasing rates of diabetes, obesity, and hypertension—have elevated stroke risk, with annual incidence estimates of around 29 per 100,000 per year and regional variations documented between 19 and 57 per 100,000 [5, 6, 7]. The Al‐Baha region, located in southwestern Saudi Arabia, presents unique public health challenges due to its mountainous terrain, dispersed rural settlements, and limited healthcare infrastructure, which restrict access to timely stroke care [8]. These factors may influence stroke outcomes and access to rehabilitation services differently compared to other regions of the country. Furthermore, extended family structures common in Al‐Baha provide substantial emotional and caregiving support, though they may also place disproportionate burdens on women caregivers. Socioeconomic disparities in income and education, combined with limited healthcare resources, further influence access to rehabilitation services and recovery trajectories [3].

Rehabilitation is a cornerstone of post‐stroke recovery, aiming to restore functional independence and improve QoL. Tools such as the EQ‐5D‐3L and the Modified Rankin Scale (mRS) are widely used to evaluate health‐related QoL (HRQoL) and functional outcomes, providing essential insights into the efficacy of rehabilitation strategies [9, 10]. However, limited data exist on the application of these measures in Saudi Arabia, particularly in underrepresented regions such as Al‐Baha. Previous studies have examined stroke incidence and outcomes in Aseer [5] and Alotaibi [6], but no dedicated research has yet addressed the Al‐Baha region. This study aims to bridge this gap by assessing the QoL and functional outcomes of post‐stroke patients in Al‐Baha, exploring associations with demographic, clinical, and rehabilitation factors. The findings are intended to inform regional healthcare policies, enhance rehabilitation strategies, and ultimately improve recovery trajectories for stroke survivors in the community.

## 2 Methods

### 2.1 Study Population and Data Collection

This cross‐sectional study included male and female post‐stroke patients aged 18–80 years who experienced a stroke within the previous 2 years. The age range reflects the actual distribution of all stroke cases retrieved during the targeted study period, with no intentional selection based on age. This ensured that all eligible cases were included to provide a comprehensive understanding of post‐stroke outcomes in the region. Patients were identified through hospital records at King Fahad Hospital, Al‐Baha. Eligibility was confirmed by reviewing clinical records and consulting treating physicians. Data collection occurred between April 2023 and May 2024, and informed consent was obtained from all participants before enrollment. Conducted in Al‐Baha, Saudi Arabia, the population was predominantly homogeneous, comprising Saudi nationals of Arab descent, making race an insignificant variable. Employment data were not collected, as most participants (aged 51–80 years) were retired. Patients with mental illnesses, pregnant women, and those unable to communicate in Arabic or English were excluded.

Data were collected between April 2023 and May 2024 at King Fahad Hospital, Al‐Baha, Saudi Arabia. A structured questionnaire was administered in face‐to‐face interviews with eligible patients. Data collection was carried out by four trained medical students from the Faculty of Medicine, Al‐Baha University, under the direct supervision of two senior investigators.

To ensure accuracy and uniformity, the data collectors underwent a 1‐day training session covering study objectives, inclusion and exclusion criteria, content of the questionnaire, ethical considerations, and interview techniques. This training minimized interviewer bias and improved consistency in data recording. All completed forms were reviewed daily by the supervisors for completeness and accuracy before entering the data set.

### 2.2 Sample Size Calculation

The sample size was determined in line with the study objective of assessing quality of life (QoL) and functional outcomes among post‐stroke patients in Al‐Baha, and examining their associations with demographic, clinical, and rehabilitation factors. A two‐tailed test was applied to estimate the number of participants required, assuming a significance level of 0.05 and a statistical power of 0.90. Using GPower software (version 3.1.9.7), the minimum required sample size was calculated to be 112 participants. However, given the exploration nature of the study and the limited availability of data in this under‐investigated population, no stopping rule was applied, and 125 patients with complete records were ultimately included in the final analysis.

### 2.3 Survey Tool

Health‐related quality of life (HRQoL) was evaluated using the Arabic validated version of the EQ‐5D‐3L, and functional outcomes were assessed with the Modified Rankin Scale (mRS). Both instruments are widely recognized, reliable, and valid measures that have been successfully applied in Middle Eastern populations, including Saudi Arabia and Jordan [11, 12, 13]. The EQ‐5D‐3L examines HRQoL across five dimensions—mobility, self‐care, usual activities, pain/discomfort, and anxiety/depression—with each dimension rated on three levels: no problems, some problems, and extreme problems. Responses generate a health profile and index value that reflect overall quality of life, enabling both intra‐ and interpopulation comparisons [9]. The mRS provides a complementary objective assessment of functional dependence, categorizing disability on a 7‐point scale from 0 (no symptoms) to 6 (death) [10].

To ensure contextual appropriateness, a structured questionnaire was developed in Google Forms, encompassing six sections: participant eligibility screening, stroke history (time since diagnosis and recurrence), sociodemographic characteristics (gender, marital status, education, income), clinical characteristics (comorbidities and physiotherapy history), disability (mRS), and HRQoL (EQ‐5D‐3L). The questionnaire underwent expert review and pilot testing with 15 stroke patients not included in the final analysis. Feedback from this pilot study was used to refine item wording, confirm cultural and linguistic suitability, and verify feasibility. Internal consistency and clarity were confirmed during this process, after which the finalized tool was implemented across the study population. This approach enabled comprehensive assessment of both objective functional outcomes and subjective QoL perceptions, offering a holistic understanding of post‐stroke recovery.

### 2.4 Statistical Analysis

Data were analyzed using IBM SPSS Statistics for Windows, Version 21.0 (IBM Corp., Armonk, NY, USA). All analyses were two‐tailed, and a *p*‐value < 0.05 was considered statistically significant. Descriptive statistics (frequencies, percentages, means, and standard deviations) were used to summarize sociodemographic, clinical, and quality‐of‐life variables. The chi‐square (*χ²*) test was applied to examine associations between categorical variables, while one‐way analysis of variance (ANOVA) was used to compare mean differences in EQ‐5D‐3L scores across groups. Cramer's *V* was calculated as a measure of effect size for chi‐square tests. Analyses were two‐tailed with a predefined significance level of *p* < 0.05, and all were pre‐specified based on the study objectives.

All analyses were conducted in accordance with the CONSORT (Consolidated Standards of Reporting Trials) guidelines to ensure transparency, reproducibility, and high‐quality statistical reporting. No missing data were encountered in this study, and all statistical terms, abbreviations, and symbols are defined upon first mention. All analyses were pre‐specified according to the study objectives; no exploratory or post‐hoc analyses were conducted.

### 2.5 Ethical Considerations

The study was conducted in accordance with the Declaration of Helsinki. Ethical approval was obtained from the Institutional Review Board of the Ministry of Health, Education, Training Center, and Academic Affairs in Al‐Baha, Saudi Arabia (Approval Number: KFH/rR8230s2023/8, approved April 2023). Written informed consent was obtained from all participants before enrollment. Anonymity and confidentiality were strictly maintained throughout the study.

## 3 Results

### 3.1 Participant Characteristics

The 125 participants were predominantly older adults (Table 1). Most individuals were married. Educational attainment varied across the sample, with a notable proportion having achieved a bachelor's degree, while others had limited formal education. Income levels predominantly fell within lower to middle brackets.

**Table 1:** Sociodemographic, clinical and comorbidity characteristics of post‐stroke participants.

Sociodemographic	*N*	%	Clinical and Comorbidity	N	%
**All**	125	100	**Time since diagnosis of stroke (Months)**		
**Sex**			1–6	11	9
Male	70	56	6–12	14	11
Female	55	44	12–18	31	25
**Age (Years)**			18–24	69	55
18–30	7	6	**Frist time incidence of Stroke**		
31–50	36	28	Yes	96	77
51–80	82	66	No	29	23
**Marital status**			**Stroke recurrence**		
Married	113	90	Yes	29	23
Single	5	4	No	96	77
Divorced	1	1	**Patients received physiotherapy**		
Widow	6	5	Yes	37	30
**Education**			No	88	70
Uneducated	34	27	**Presence of chronic diseases**		
Primary school	25	20	Yes	100	80
Elementary school	16	13	No	25	20
Secondary school	13	10	**Diabetes**		
Bachelor's degree	37	30	Yes	71	57
**Monthly Income (SAR)**			No	54	43
< 5 K	73	59	**Hypertension**		
5–10 K	39	31	Yes	71	57
> 10 K	13	10	No	54	43
			**Other comorbidities**		
			Yes	15	12
			No	110	88

### 3.2 Clinical Characteristics and Comorbidities

The clinical profiles of the study participants reflect the multifaceted nature of post‐stroke recovery, with varying durations from diagnosis, stroke recurrence, comorbidities, and rehabilitation practices (Table 1). A significant proportion of participants had experienced their first stroke, with a smaller subset reporting recurrent episodes. The cohort exhibited a high prevalence of chronic diseases, notably diabetes and hypertension.

### 3.3 Disability Outcomes Assessed by the Modified Rankin Scale (mRS)

The mRS revealed that 59% of participants achieved favorable outcomes, while 41% experienced moderate to severe disability or death (Table 2). A statistically significant association was observed between age and mRS outcomes (*χ²*(2, *N* = 125) = 13.96, *p* = 0.003, Cramer's *V* = 0.33), indicating a moderate effect size. Younger participants (18–30 years) achieved exclusively favorable outcomes (100%, *n* = 7), while older participants, particularly those aged 51–80 years, showed higher rates of poor outcomes (52%, *n* = 43). Gender was not significantly associated with mRS outcomes (*χ²* (1, *N* = 125) = 0.40, *p* = 0.527, *V* = 0.06). Females reported 58% favorable outcomes compared to 57% among males. Physiotherapy utilization showed a statistically significant association with mRS outcomes (*χ²* (1, *N* = 125) = 3.97, *p* = 0.047, *V* = 0.18), though the effect size was small. Participants who received physiotherapy had a higher proportion of poor outcomes (57%, *n* = 21) compared to those who did not (46%, *n* = 32). Stroke recurrence showed a nonsignificant trend toward poorer outcomes (*χ²*(1, *N* = 125) = 2.97, *p* = 0.085, *V* = 0.15). Poor outcomes were observed in 55% (*n* = 16) of those experiencing recurrent strokes compared to 38% (*n* = 37) among participants without recurrence.

**Table 2:** Distribution of modified Rankin scale (mRS) scores among participants.

	Best outcomes			Poor Outcomes				
	No symptoms at all	No significant disability despite signs: able to carry out all usual duties and activities.	Slight disability: unable to carry out all previous activities, but able to look after own affairs without assistance	Moderate disability: requiring some help, but able to walk without assistance	Moderately severe disability: unable to walk without aid and unable to attend to own bodily needs without assistance	Severe disability: bedridden, incontinent, and requiring constant nursing care and attention	Dead	*p* value
	22 (18%)	28 (23%)	22 (18%)	23 (18%)	13 (10%)	14 (10%)	3 (2%)	
**All**	72 (59%)			53 (41%)				
**Age**								
18–30	7 (100%)			0 (0%)				0.003
31–50	26 (72%)			10 (28%)				
51–80	39 (48%)			43 (52%)				
**Sex**								
Female	32 (58%)			23 (42%)				0.527
Male	40 (57%)			30 (43%)				
**Patients received physiotherapy**								
Yes	16 (43%)			21 (57%)				0.047
No	56 (64%)			32 (46%)				
**Stroke recurrence**								
Yes	13 (45%)			16 (55%)				0.085
No	59 (62%)			37 (38%)				

### 3.4 Quality of Life Assessment Using Eq‐5D‐3L

**Figure 1:**
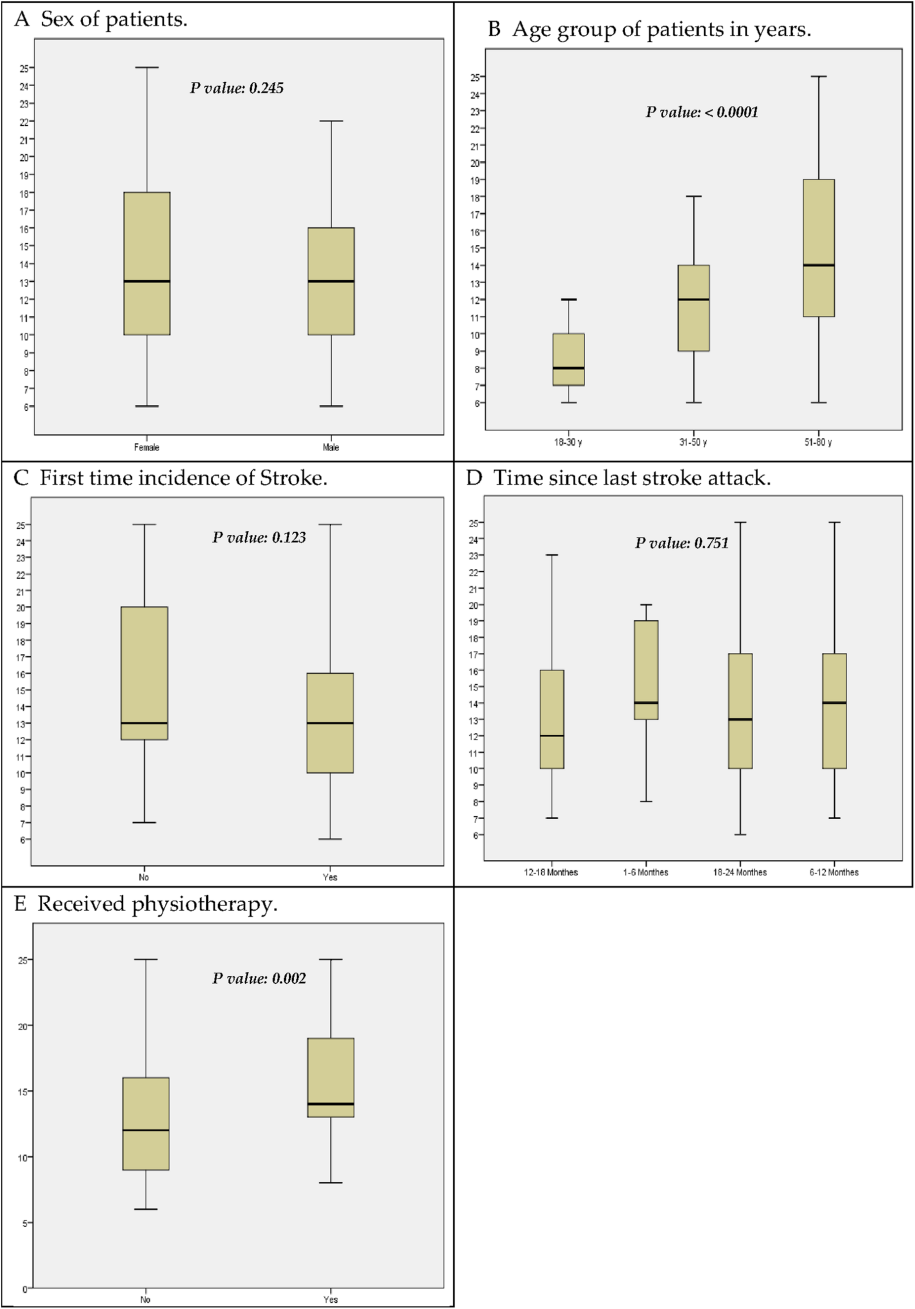
Aggregate Quality of Life Scores Post‐Stroke Patients. Pooled QoL (Quality of life) was calculated by adding scores from different variables including mobility, self‐care, usual activities, pain/discomfort, anxiety/depression and comorbidities, the pooled score ranged between 5 and 29, the quality of life for patients gets worse as the score get closer to the upper range of the pooled score. A. Sex of patients. B. Age group of patients in years. C. First time incidence of Stroke. D. Time since last stroke attack. E. Received physiotherapy.

The EQ‐5D‐3L assessed quality of life (QoL) across five dimensions: mobility, self‐care, usual activities, pain/discomfort, and anxiety/depression. Pooled scores ranged from 5 to 29, with a mean ± SD of 14 ± 4.57, as illustrated in Figure 1. As shown in Table 3, self‐rated health was categorized as *poor* (19%), *intermediate* (36%), and *best* (45%), with significant differences in mean scores (*poor*: 24 ± 7; *best*: 83 ± 10, *p* < 0.001). A significant association was observed between age and self‐rated health (*χ²*(4, *N* = 125) = 7.32, *p* = 0.025, Cramer's *V* = 0.17), indicating a small effect size. Younger participants (18–30 years) reported “best” health in 57% of cases compared to 37% among those aged 51–80 years. No significant associations were found for gender, physiotherapy, or stroke recurrence (*p* > 0.05). Mobility and pain/discomfort were the most affected dimensions (58% and 64%, respectively), while self‐care showed minimal impairment (39%). Anxiety/depression was unproblematic for half of the participants (Tables 4 and 5).

**Table 3:** Summary of responses to self‐rated health status.

	All	Poor	Intermediate	Best	*p*‐value
***N* (%)**	125 (100%)	24 (19%)	45 (36%)	56 (45%)	
**Mean ± SD**	60 ± 24	24 ± 7	50 ± 9	83 ± 10	< 0.0001
**Median (IQR)**	60 (40–80)	30 (20–30)	50 (40–60)	80 (70–90)	< 0.0001
**Age**					
18–30	7 (6%)	0 (0%)	3 (43%)	4 (57%)	0.025
31–50	36 (28%)	2 (6%)	12 (33%)	22 (61%)	
51–80	82 (66%)	22 (27%)	30 (37%)	30 (37%)	
**Sex**					
Female	55 (44%)	14 (26%)	19 (35%)	22 (40%)	0.277
Male	70 (56%)	10 (14%)	26 (37%)	34 (49%)	
**Patients received physiotherapy**					
Yes	88 (70%)	7 (19%)	16 (43%)	14 (38%)	0.531
No	37 (30%)	17 (19%)	29 (33%)	42 (48%)	
**Stroke recurrence**					
Yes	29 (23%)	7 (24%)	12 (41%)	10 (35%)	0.432
No	96 (77%)	17 (18%)	33 (34%)	46 (48%)	

**Table 4:** Quality of life scores based on EQ‐5D‐3L dimensions.

	Mobility	Selfcare	Usual activities	Pain/Discomfort	Anxiety/Depression	Pooled score†			
							*N* (%)	Mean ± SD	*p* value
**Level 1**	52 (42%)	76 (61%)	58 (46%)	45 (36%)	62 (50%)	**All**	125 (100%)	14 ± 4.57	
						**Best QoL**	57 (45%)	10 ± 1.84	< 0.001
**Level 2**	58 (46%)	26 (21%)	38 (30%)	34 (27%)	63 (50%)	**Intermediate QoL**	42 (34%)	14 ± 1.46	
**Level 3**	15 (12%)	23 (18%)	29 (24%)	46 (37%)	0 (0%)	**Poor QoL**	26 (21%)	20 ± 2.06	

Abbreviations: EQ‐5D‐3L – EuroQol 5‐Dimension 3‐Level. Dimensions include mobility, self‐care, usual activities, pain/discomfort, and anxiety/depression, with each scored on three levels: no problems, some problems, and extreme problems.

^†^ Pooled QoL (Quality of life) score was calculated by adding scores from several variables including mobility, self‐care, usual activities, pain/discomfort, anxiety/depression and comorbidities, the pooled score ranged between 5 and 29, the quality‐of‐life score for patients get worse as the pooled score get closer to the upper range. P‐values were calculated using the ANOVA test.

**Table 5:** Quality of life analysis stratified by key variables.

Disability		Age			*p* value	Sex		*p* value	Received physiotherapy		*p* value	Stroke recurrence		*P* value
		18–30	31–50	51–80		Female	Male		Yes	No		Yes	No	
**Mobility**	L1	6 (11%)	17 (33%)	29 (56%)	0.007	25 (48%)	27 (53%)	0.06	46 (89%)	6 (11%)	0.001	45 (86%)	7 (14%)	0.022
	L2	1 (2%)	19 (33%)	38 (66%)		20 (35%)	38 (65%)		34 (59%)	24 (41%)		43 (74%)	15 (26%)	
	L3	0 (0%)	0 (0%)	15 (100%)		10 (67%)	5 (33%)		8 (53%)	7 (47%)		8 (53%)	7 (47%)	
**Selfcare**	L1	7 (9%)	27 (36%)	(55%)	0.005	35 (46%)	41 (54%)	0.005	59 (78%)	17 (22%)	0.085	62 (82%)	14 (18%)	0.039
	L2	0 (0%)	8 (31%)	18 (69%)		5 (19%)	21 (81%)		15 (58%)	11 (42%)		21 (81%)	5 (19%)	
	L3	0 (0%)	1 (4%)	22 (96%)		15 (65%)	8 (35%)		14 (61%)	9 (39%)		13 (57%)	10 (43%)	
**Usual Activities**	L1	7 (12%)	22 (38%)	29 (50%)	0.001	24 (41%)	34 (59%)	0.064	46 (79%)	12 (21%)	0.029	48 (83%)	10 (17%)	0.091
	L2	0 (0%)	11 (29%)	27 (71%)		13 (34%)	25 (66%)		27 (71%)	11 (29%)		30 (79%)	8 (21%)	
	L3	0 (0%)	3 (10%)	26 (90%)		18 (62%)	11 (38%)		15 (52%)	14 (48%)		18 (62%)	11 (38%)	
**Pain/Discomfort**	L1	4 (9%)	17 (38%)	24 (53%)	0.161	14 (31%)	31 (69%)	0.004	36 (80%)	9 (20%)	0.033	34 (76%)	11 (24%)	0.354
	L2	0 (0%)	8 (24%)	26 (76%)		12 (35%)	22 (65%)		26 (76%)	8 (24%)		29 (85%)	5 (15%)	
	L3	3 (7%)	11 (24%)	32 (69%)		29 (63%)	17 (37%)		26 (56%)	20 (44%)		33 (72%)	13 (28%)	
**Anxiety/Depression**	L1	4 (7%)	18 (29%)	40 (65%)	0.912	23 (37%)	39 (63%)	0.086	47 (76%)	15 (24%)	0.123	47 (76%)	15 (24%)	0.480
	L2	3 (5%)	18 (29%)	42 (67%)		32 (51%)	31 (49%)		41 (65%)	22 (35%)		49 (78%)	14 (22%)	
	L3	0 (0%)	0 (0%)	0 (0%)		0 (0%)	0 (0%)		0 (0%)	0 (0%)		0 (0%)	0 (0%)	
**Pooled score**	Poor	0 (0%)	1 (4%)	25 (96%)	< 0.001	15 (58%)	11 (42%)	0.137	14 (54%)	12 (46%)	0.006	15 (58%)	11 (42%)	0.033
	Intermediate	0 (0%)	14 (33%)	28 (67%)		14 (33%)	28 (67%)		26 (62%)	16 (38%)		35 (83%)	7 (17%)	
	Best	7 (12%)	21 (37%)	29 (51%)		26 (46%)	31 (54%)		48 (84%)	9 (16%)		46 (81%)	11 (19%)	

Abbreviations: EQ‐5D‐3L – EuroQol 5‐Dimension 3‐Level. Dimensions include mobility, self‐care, usual activities, pain/discomfort, and anxiety/depression, each scored on three levels: no problems, some problems, and extreme problems. *P*‐values were calculated using the chi‐square test.

## 4 Discussion

This study aimed to assess the quality of life (QoL) and functional outcomes of post‐stroke patients in Al‐Baha, Saudi Arabia, and to explore their associations with demographic, clinical, and rehabilitation factors. Overall, the findings reveal that a substantial proportion of participants experienced moderate to severe disability and reported impairments in multiple QoL dimensions, particularly mobility and pain/discomfort. These outcomes were significantly influenced by age and stroke recurrence, while gender showed no significant effect. Physiotherapy use was associated with functional status, likely reflecting baseline severity rather than treatment effect.

The functional outcomes, as measured by the Modified Rankin Scale (mRS), revealed that 41% of participants experienced moderate to severe disability or death. This finding underscores the substantial proportion of survivors requiring ongoing support to achieve greater independence and underscores the importance of timely and targeted interventions [14]. Conversely, 59% of participants achieving favorable outcomes indicate the potential for recovery when rehabilitation services are accessible and effectively utilized. However, disparities in rehabilitation access between urban and rural areas in Al‐Baha remain a significant barrier, necessitating strategies to enhance service availability. Age was a critical determinant of recovery outcomes, with younger participants reporting significantly better QoL and functional independence compared to older age groups. This finding is consistent with global trends, as younger individuals generally exhibit greater physiological resilience and a lower prevalence of comorbidities [15, 16]. In Al‐Baha, the higher prevalence of chronic conditions such as diabetes and hypertension among older populations further complicates recovery [17, 18]. These results suggest that tailored interventions, including early screening and integrated management of comorbidities, are essential to improve recovery trajectories for older adults.

Physiotherapy utilization was significantly associated with functional outcomes in this study; however, patients who received physiotherapy exhibited poorer outcomes compared to those who did not. This observation is likely attributable to selection bias, wherein individuals with more severe initial disabilities were more frequently referred for rehabilitation services. Thus, the observed association may reflect greater baseline impairment rather than the direct effect of physiotherapy itself. Nonetheless, physiotherapy remains an essential component of stroke rehabilitation, with extensive evidence supporting its role in promoting recovery and improving quality of life. Future prospective studies, incorporating adjustment for stroke severity and other potential confounders, are warranted to more accurately elucidate the true impact of physiotherapy on functional recovery and quality of life in stroke survivors. In Al‐Baha, the effectiveness of physiotherapy is further constrained by limited access to qualified therapists and inconsistent rehabilitation protocols. These findings align with studies conducted in other Middle Eastern regions, such as Alhazzani et al. (2018) [2] and Alotaibi et al. (2021) [10], emphasizing the role of socioeconomic and geographic factors in stroke recovery outcomes. Furthermore, the limited impact of physiotherapy on QoL scores observed in our study mirrors findings from rural communities globally where access to multidisciplinary care is constrained [5, 19]. To reflect the integration of rehabilitation into the study's objectives, the title was revised to emphasize the role of rehabilitation [20, 21]. Addressing these challenges requires expanding physiotherapy services, introducing tele‐rehabilitation programs to overcome geographic barriers, and integrating multidisciplinary approaches to address both physical and emotional aspects of recovery. Combining physiotherapy with chronic disease management and psychological support could enhance overall outcomes and QoL for stroke survivors.

The EQ‐5D‐3L results showed that participants reported impairments across multiple domains, with mobility and pain/discomfort most affected. Self‐rated health varied significantly, with younger participants reporting better outcomes compared to older groups. No significant differences were observed by gender, physiotherapy use, or stroke recurrence. These impairments align with global research findings but may be exacerbated in Al‐Baha due to the region's unique geographic, socioeconomic, and healthcare challenges [10, 11, 12]. Limited access to specialized rehabilitation services, coupled with the logistical difficulties faced by patients in rural areas, likely contributes to these adverse outcomes [13, 14, 15]. The study offers actionable insights to enhance rehabilitation strategies, particularly in underserved regions like Al‐Baha, and emphasizes the importance of addressing these challenges to improve recovery trajectories for stroke survivors.

Recurrent strokes were strongly associated with poorer outcomes, emphasizing the importance of secondary prevention measures. Effective prevention strategies, including lifestyle modifications, medication adherence, and regular follow‐ups, are crucial in reducing recurrence risk [22]. In the context of Al‐Baha, community‐based education programs are particularly valuable. Collaborations with local mosques, schools, and community centers could improve public awareness of stroke risk factors and promote adherence to preventive measures [23]. Engaging primary healthcare providers in post‐stroke care could further enhance the continuity and effectiveness of these prevention efforts.

This study's strength lies in its use of validated tools such as the EQ‐5D‐3L and the Modified Rankin Scale (mRS), which provide reliable assessments of QoL and functional outcomes [1, 24]. Additionally, the study's focus on a relatively underrepresented region, Al‐Baha, contributes valuable data to the limited body of literature on stroke recovery in Saudi Arabia. However, direct local validation of these tools in the Al‐Baha region was not performed. Given the similarity of the target population to surrounding regions where these tools have been validated, we considered them appropriate for use. Future research could explore localized validation to further strengthen their applicability. Despite employing robust statistical methods, the study has limitations. Its cross‐sectional design precludes causal inferences, and the small sample size limits generalizability. The reliance on self‐reported QoL data introduces potential response bias, particularly in culturally sensitive domains such as anxiety and depression. Future research should adopt longitudinal designs to capture long‐term recovery trajectories and include larger, multicenter samples to enhance the representativeness of findings. Detailed data on the type, frequency, and duration of rehabilitation interventions could also provide deeper insights into the factors influencing recovery outcomes.

## 5 Conclusions

This study highlights the significant impact of stroke on quality of life and functional outcomes among patients in Al‐Baha, Saudi Arabia. Findings from the EQ‐5D‐3L and Modified Rankin Scale reveal moderate impairments, with mobility and pain/discomfort as the most affected dimensions. Younger participants demonstrated better outcomes, while older age, recurrent strokes, and comorbidities were associated with greater disability and poorer quality of life. Physiotherapy showed limited impact on outcomes, underscoring the need for further research into its optimization. These results emphasize the importance of targeted, individualized rehabilitation strategies to address specific impairments and improve recovery trajectories in post‐stroke populations.

## Author Contributions

Y.M. Kofiah and T. Alkully conceptualized the study, supervised the research, and critically revised the manuscript. U. Albakri, Batol M. Albanghali Oyhman, B.M. Albanghali, Haya A. Alzahrani Alzahrani, S.M. Alghamdi, A.N. Alzahrani, and Rana A. Algahamdi Algahamdi collected and analyzed the data. A.M. Alzahrani contributed to statistical analysis and technical expertise. Mohammad A. Albanghali Albanghali coordinated the study, drafted the manuscript, and approved the final submission. All authors have read and approved the final version of the manuscript. The corresponding author had full access to all the data in this study and takes complete responsibility for the integrity of the data and the accuracy of the data analysis.

## Institutional Review Board Statement

The study was conducted in accordance with the Declaration of Helsinki and approved by the Institutional Review Board of the Ministry of Health, Education, Training Center, and Academic Affairs in Al‐Baha, Saudi Arabia (Approval Number: KFH/rR8230s2023/8, approved in April 2023).

## Consent

Informed consent was obtained from all subjects involved in the study.

## Conflicts of Interest

The authors declare no conflicts of interest.

## Transparency Statement

The lead author Mohammad A. Albanghali affirms that this manuscript is an honest, accurate, and transparent account of the study being reported; that no important aspects of the study have been omitted; and that any discrepancies from the study as planned (and, if relevant, registered) have been explained.

## Data Availability

The datasets used and/or analyzed in this study are available from the corresponding author upon reasonable request.
